# Seeing the Forest but Naming the Trees: An Object-Over-Place Bias in Learning Noun Labels

**DOI:** 10.1162/opmi_a_00154

**Published:** 2024-08-09

**Authors:** Yi Lin, Moira R. Dillon

**Affiliations:** Department of Psychology, New York University, New York, USA

**Keywords:** word learning, objects, places, geometry, syntax, labels, nouns, prepositions

## Abstract

Objects and places are foundational spatial domains represented in human symbolic expressions, like drawings, which show a prioritization of depicting small-scale object-shape information over the large-scale navigable place information in which objects are situated. Is there a similar object-over-place bias in language? Across six experiments, adults and 3- to 4-year-old children were asked either to extend a novel noun in a labeling phrase, to extend a novel noun in a prepositional phrase, or to simply match pictures. To dissociate specific object and place information from more general figure and ground information, participants either saw scenes with both place information (a room) and object information (a block in the room), or scenes with two kinds of object information that matched the figure-ground relations of the room and block by presenting an open container with a smaller block inside. While adults showed a specific object-over-place bias in both extending novel noun labels and matching, they did not show this bias in extending novel nouns following prepositions. Young children showed this bias in extending novel noun labels only. Spatial domains may thus confer specific and foundational biases for word learning that may change through development in a way that is similar to that of other word-learning biases about objects, like the shape bias. These results expand the symbolic scope of prior studies on object biases in drawing to object biases in language, and they expand the spatial domains of prior studies characterizing the language of objects and places.

## INTRODUCTION

Objects and places are foundational spatial domains that support the commonsense intelligence and behavior of humans and nonhuman animals alike. Even from early in development, humans and nonhuman animals treat objects and places differently, including during daily cognitive tasks like recognition and navigation (Dillon et al., 2018; Doeller & Burgess, 2008; Doeller et al., 2008; Epstein & Kanwisher, 1998; Julian et al., 2015). This differential treatment of objects and places is supported by different underlying mental representations and brain structures, which have been studied for decades at various levels of analysis from neurons to behavior (see Spelke & Lee, 2012 for a review).

In humans, different representations of objects and places also play out in surprising and unique ways, for example, by affecting our early symbolic expressions, like our use of pictures (Dillon & Spelke, 2015, 2017). In particular, young children’s spontaneous drawings suggest that they tend to draw mostly small-scale objects, not the large extended place information in which those objects are situated (Machón, 2013; Piaget & Inhelder, 1967). Empirical investigations probing children’s drawings of such object and place information when children are given a model with both types of information have found, moreover, that up to 90% of children omit place information entirely from their drawings (Ebersbach et al., 2011; Lange-Küttner, 2014). Anthropologists and art historians too have noted the surprising lack of place information in the art of human prehistory, with a robust prioritization of object information (Clottes, 2008).

Until recently, this object-over-place bias in human drawings could have been attributed to a more general figure-over-ground bias: In daily life, objects are more likely to be figure information in the foreground of a scene while places are more likely to be ground information in the background of a scene. To address whether children show a domain-specific object bias or a more general figure bias in drawing, Dillon (2021) presented 4-year-old children with a drawing production task in which they were asked to draw “exactly what they saw” while either sitting in a colorful rectangular room with rectangular objects inside or sitting in front of a toy, object-model of the room, which matched the room in its figure-ground and size relations. Children’s drawings often omitted the ground (i.e., the walls) that composed the room’s large-scale place information but included the corresponding ground (i.e., object-parts) for the toy. This work thus proposed a bias in young children’s drawings rooted specifically in spatial domain: Young children’s drawings reflect an object-over-place bias.

The present work asks whether a similar object-over-place bias exists in humans’ first symbolic expressions: language. Do young children show an object-over-place bias in word learning? For decades, researchers have charted a number of intuitions and biases that toddlers and young children rely on when learning new words (e.g., Landau et al., 1988; Markman & Hutchinson, 1984; Nelson, 1988). For example, when toddlers hear a novel noun label applied to an object (e.g., “This is a *dax*!”), they often generalize that noun (e.g., “Which one of these is a *dax*?”) to another object of the same shape as the labeled object rather than to an object of the same color or texture (Landau et al., 1988), a tendency called the “shape bias.” This bias is reliable by around 2.5-years-old (Hupp, 2008) but may be elicited more or less strongly based on the labeled object’s function, complexity, or topology (Cimpian & Markman, 2005; Kenderla et al., 2023; Nelson et al., 2017; Smith et al., 1996), as well as toddlers’ linguistic ability (Gershkoff-Stowe & Smith, 2004; Jones, 2003) or conceptual knowledge about the labeled object (Booth & Waxman, 2002; Waxman, 1994). The shape bias joins other word-learning intuitions and biases, including a whole-object bias, in which toddlers generalize a noun to whole objects as opposed to object parts (Hollich et al., 2007; Markman & Wachtel, 1988), and a basic-level-category bias, in which toddlers generalize a noun to an object’s basic-level category as opposed to its superordinate or subordinate category (Golinkoff et al., 1995). These biases are all elicited in toddlers and young children in word-learning contexts with novel noun labeling phrases more reliably than in simple matching contexts or in contexts in which only anaphoretic language is used (e.g., “Which *one* matches this *one*?”).

Different, nonlabeling syntactic frames, moreover, elicit word learning about other parts of speech. For example, children and adults tend to extend novel adjectives (e.g., “This one is *glozen*.”) by focusing on a sample object’s properties, like its color or texture (Waxman & Markow, 1998). And, they tend to extend novel prepositions (e.g., “This is *acorp* my box.”) by focusing on a sample object’s location or “place” on a ground object (Fisher et al., 2006; Landau & Stecker, 1990). Indeed, Landau and Jackendoff (1993) outline the importance of considering such object and place information in spatial language, spatial cognition, and the relation between the two. Their account describes how language reflects objects’ and places’ nonlinguistic spatial properties, like their shapes, sizes, and figure-ground relations. Landau and Jackendoff (1993) nevertheless propose a specific definition of “places” that relies on spatial relations between objects only—objects like *cat*, *mat*, *book*, *table*, *chair*, *bicycle*, and *house*. Other linguistic descriptions of “places,” such as those conveyed by spatial verbs (e.g., *support*, *hold*, *contain*) or nouns (e.g., *table*, *statue*, *boat*) similarly focus on places as relations between objects (e.g., Bennett, 1993; Landau, 2017; Tversky & Clark, 1993). While such objects in these descriptions may be animate (e.g., *cat*) or large and fixed (e.g., *house*), this prior work nevertheless still focused primarily on the domain of objects or treated objects and places in an undifferentiated way, not fully accounting for their distinguishing geometric features. That is, we suggest that while objects tend to have a particular bounded shape, be free-standing, and be potentially manipulable, places are instead delineated by the fixed geometry of a large-scale extended surface layout and are navigable (Lee et al., 2006). And so, while the present work follows in the tradition of Landau and Jackendoff (1993) in its emphasis on the importance of accounting for nonlinguistic differences between objects and places in language, it extends their argument to capture a different and foundational definition of places that captures places’ specific geometric features. For example, while previous work would have considered “The *book* is on the *table*.” as language relating an object (*book*) to a place (*table*; Landau & Jackendoff, 1993; see also Landau, 2017; Landau & Stecker, 1990; Talmy, 1978), in the present work, both *book* and *table* would be considered objects. But, “The *book* is in the *room*.” would be considered a sentence about an object in a place. With our definition of places, we are able to thereby examine how objects and places, with their distinctive set of core geometric features (Lee et al., 2006; Spelke, 2022; Spelke & Lee, 2012), might relate to one another and confer their own, specific set of biases in word learning.

In the present set of six experiments, we thus tested whether there is a specific object-over-place bias in word learning. The first three experiments tested adults. In Experiment 1A, adults were randomly assigned to either a *place condition* or an *object condition*. In the *place condition*, adults saw a rendered cartoon picture of a room of a particular shape (e.g., a dome-shaped room) with a block of a different shape inside it (e.g., a hexagonal block). The scene was labeled with a novel noun, and adults were asked to extend this novel noun to another scene either with its same place information (i.e., room shape) or with its same object information (i.e., block shape). To dialogue directly with and examine the spatial scope of prior studies that focused on the shape bias and other such biases about objects (e.g., Landau et al., 1988), we used the same kind of labeling phrases as in those studies, e.g., “Here is a *blicket*! Where is another *blicket*?” We predicted that adults would extend the novel noun label to the object information in the sample scene, suggesting an object bias in extending noun labels.

Since object and place information was confounded with other more general spatial information like figure-ground and size relations in prior studies and in the *place condition* (where the block was always in the foreground of the scene and the room was always in the background of the scene), the *object condition* in Experiment 1A was critical to examining the specificity of any object bias to object information. In the *object condition*, adults were thus asked to extend a novel noun label to either one of two objects, one in the foreground of the scene and one in the background of the scene. To match the figure-ground and size relations presented in the place condition, the background object was presented as an open container/diorama with the foreground object inside. If, as predicted, adults did not preferentially extend the novel noun label to the smaller foreground object in the object condition, then their extension of the noun label to the smaller foreground object in the place condition would be due to spatial category alone, not to more general spatial information, like figure-ground or size relations.

Experiments 1B, 2, and 3 further explored the role of spatial information and syntactic framing in adults’ object bias, found in Experiment 1A. Experiment 1B ruled out the possibility that adults in the place condition of Experiment 1A were actually extending the novel noun label based on place information, but were doing so by relying on a place’s object contents, not its global shape (i.e., participants might have thought that *blickets* were places that have certain kinds of objects in them, like *kitchen*s are places that have refrigerators in them). Experiments 2 and 3 then examined whether adults’ object bias in Experiment 1A was specific to their extension of novel noun labels, whose syntactic framing may more readily convey object information, compared with the syntactic framing of simple matching phrases (Experiment 2; Landau et al., 1988) or novel prepositional phrases (Experiment 3; Landau & Stecker, 1990). Adults showed the same pattern of results in Experiment 2, suggesting that their object bias was not specific to word-learning contexts. However, they showed a different pattern of results in Experiment 3, suggesting that their object bias was nevertheless stronger in response to labeling versus prepositional phrases. In particular, and consistent with prior work (Fisher et al., 2006; Landau & Stecker, 1990), the prepositional phrases in Experiment 3 encouraged more noun extensions to place information than did labeling phrases. Nevertheless, prepositional phrases also appeared to encourage a more general figure-over-ground bias, which is potentially inconsistent with prior work (Landau & Jackendoff, 1993).

To examine if young children might show the same pattern of results as adults, we conducted the same set of experiments with children in Experiments 4–6. Previous research has found both that word-learning biases often get stronger with age (Landau et al., 1988, 1992) and that biases shown in word-learning contexts often extend to nonword-learning contexts, e.g., matching contexts, with age (Landau et al., 1988). We explored both of these possibilities. We chose to focus on 3- and 4-year-old children for two reasons. First, our experiments used shape as the basis for extending nouns to object or place information, and children at this age are old enough to show a strong shape bias for objects (Landau et al., 1988, 1992; Landau & Stecker, 1990). Second, our experiments probed an understanding of the spatial relations encoded by prepositional phrases and in sentences with two novel words. Children at this age can flexibly understand syntax with novel nouns or prepositions as well as sentences with two novel words (Landau & Stecker, 1990; White & Lidz, 2022). While children showed the same object bias as adults when extending noun labels (Experiment 4), their responses showed a less clear pattern for the matching context (Experiment 5). Moreover, while, like adults, prepositional phrases encouraged more noun extensions to place information than did labeling phrases, it was unclear how children interpreted these novel prepositional phrases (Experiment 6).

Overall, our experiments reveal an object-over-place bias in the understanding of novel labeling phrases from at least young childhood as well as a transfer of that bias to nonword-learning, matching, contexts by at least adulthood. Our experiments also suggest that this bias is not present when we interpret novel nouns as the referents of novel prepositions. Our findings happen in the context of a definition of places that considers their core and unique geometric properties as large, extended, and navigable. Together, our results expand the symbolic scope of prior studies on object biases in drawing to object biases in language, and they expand the spatial domains of prior studies characterizing the language of objects and places.

## EXPERIMENT 1A

### Methods

All of the experiment’s methods were preregistered prior to data collection.

#### Participants.

Seventy-two adults (*M*age: 19 years; range: 18–22 years; 51 women, 19 men, 2 gender nonbinary) constituted our sample, with 36 adults in each of two conditions. All were native English speakers, were recruited from our university’s participant pool, and received course credit for their participation. An additional 12 adults participated in the experiment but were excluded following preregistered criteria: for taking longer than 15 minutes to complete the experiment (3); or for answering at least one catch question incorrectly (9; see below). The use of human participants for this experiment was approved by the institutional review board on the use of human subjects at our university.

#### Stimuli.

**Figure 1.**
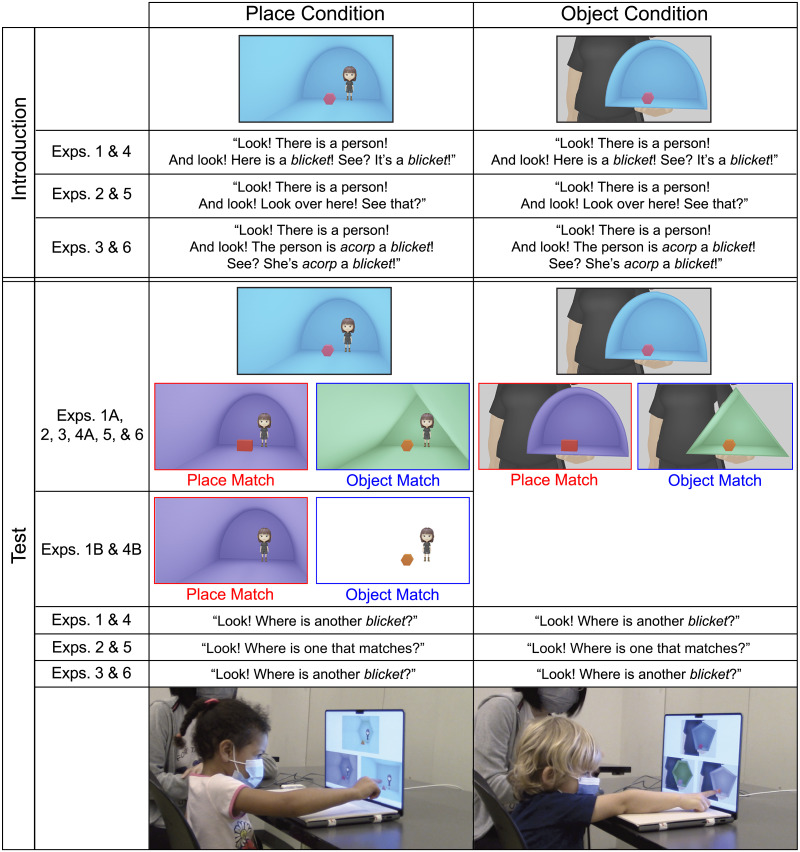
Schematic of the experimental stimuli and procedures. Experiments 1– 3 were with adults, and Experiments 4–6 were with children. In Experiments 1, 3, 4, and 6, participants heard a novel noun applied ambiguously to the contents of a sample picture and were asked to extend that noun. In Experiments 1 & 4, the noun was presented in a labeling phrase, and in Experiments 3 & 6, the noun was presented in a prepositional phrase. In Experiments 2 & 5, participants heard no noun label but were asked to match pictures. To dissociate object and place information from figure and ground information in each experiment, participants were assigned to one of two conditions. In the *place condition*, participants saw place information (a room of a particular shape) and object information (a block of a particular shape inside the room). In the *object condition*, by contrast, participants saw two kinds of objects, one in the foreground of the scene and one in the background of the scene, matching the figure-ground and size relations presented in the place condition by presenting a kind of open container/diorama with a smaller object inside. Experiments 1B & 4B presented only the place condition and showed isolated place or object information in the test-trial scenes.

The stimuli consisted of a set of 2D place pictures with 72 3D rendered scenes composed of place and object information and generated in the animation software Blender (Figure 1, Experiment 1A, Place Condition). To focus on a difference in spatial category, we used rendered, as opposed to natural, scenes, which not only allowed us to control for visual features like color and texture, which may differ between place and object information in typical natural and man-made scenes, but also allowed us to instantiate the very same basic shapes as either place or object information.

After Dillon et al. (2018), the place information was conveyed as a room of one of eight shapes: rectangle; dome; isosceles triangle; regular hexagon; square; elongated hexagon; elongated pentagon; or elongated parallelogram and was shown in a cool color: blue; purple; or green. To best demonstrate that the room was navigable, a 3D rendered cartoon person stood by the room’s back wall. Each picture also included one object, conveyed as a 3D block, whose shape was chosen from the same set of eight shapes as the room but presented a different shape from the room. The block was shown in a warm color: pink; red; or orange. The full set of 72 place pictures fully permuted the shape of the room with the shape of the block in it, with three examples of each room/block pair, which varied in color.

Two trials were presented to each participant, which included completely different shapes, colors, and color pairings. One trial included the rectangle, dome, isosceles triangle, and regular hexagon. The other trial included the square, elongated hexagon, elongated pentagon, and elongated parallelogram. Different shades of the six colors were also used in the two trials. Each of the two trials presented a sample picture (e.g., a dome-shaped room with a hexagonal block inside), a place-match picture with the same-shaped room as the room in the sample picture but with a different-shaped block (e.g., a dome-shaped room with a rectangular block inside) and an object-match picture with a same-shaped block as the block in the sample picture but with a different-shaped room (e.g., a triangular room with a hexagonal block inside; Figure 1, Experiment 1A, Place Condition).

The colors of the rooms and blocks varied across all three pictures. Across participants, each of the 36 pictures from each trial was shown once as the sample. With 36 possible pictures per trial, there were 144 possible sets of sample, place-match, and object-match pictures. Thirty-six of these 144 possible sets of sample and response pictures were presented across participants with the constraints that each shape was presented an equal number of times as the place match and the object match and each color was presented an equal number of times as the place match and the object match. In addition: the place-match picture appeared on the left and right side of the screen an equal number of times across participants; the place-match picture appeared on the same and different side of the screen across the two trials an equal number of times across participants; each pair of colors appeared in the sample, place-match, and object-match pictures an equal number of times across participants; and half of the participants were shown the place-match picture on the left side of the screen on the first trial, while half of the participants were shown the place-match picture on the right side of the screen on the first trial.

A complementary set of 72 object pictures (Figure 1, Experiment 1A, Object Condition) was also created in Blender. These pictures matched the place pictures in the shape of the figure and ground elements as well as in the size and position of these elements in the pictures themselves. Instead of presenting a room and a block, which differed in their spatial category, these pictures presented a container/diorama with its open side forward and with a closed block placed inside. The same cartoon person from the place pictures was now shown holding the container. The figure and ground elements thus both presented object information. Trials using these pictures also used a sample picture, place-match (i.e., ground-match) picture, and object-match (i.e., figure-match) picture with all the same variation and counterbalancing across trials and participants.

#### Procedure.

Participants completed the experiment online through the survey platform Qualtrics and without interacting with an experimenter. Participants first read instructions telling them how to view the experiment in their browser window and test their computer’s audio. Then, participants saw two test trials, as described above. The sample picture appeared at the top-center of the screen, and participants heard pre-recorded sentences describing the picture with a novel noun, “Look! There is a person! And look! Here is a *blicket*! See? It’s a *blicket*!” (Figure 1, Experiment 1A). The person in the picture was explicitly labeled to eliminate it as a potential referent of the novel noun. Then, two test pictures—a place match and an object match—appeared below the sample picture, which remained visible. Participants were asked, “Where is another *blicket*?”, and used their mouse to select one of the two response pictures. After making their response, participants answered a catch question, in which they selected which one of four novel words they just heard, given the choices: *blicket*; *snuz*; *lorp*; *wug*; or *I heard something else (Enter what you heard)*. Participants did not receive feedback on the test or catch questions. All participants heard the first sample picture labeled with the novel noun *blicket* and the second sample picture labeled with the novel noun *wug*.

### Results

**Figure 2.**
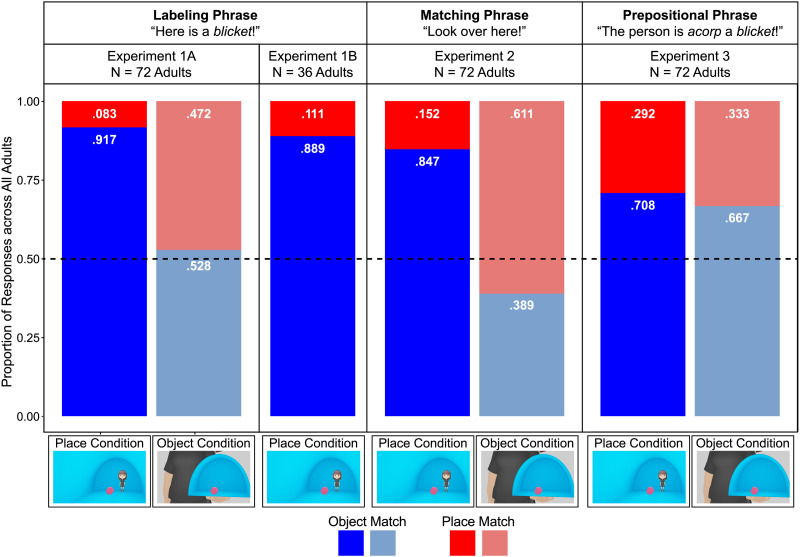
The proportion of object-match responses (in blue) and place-match responses (in red), across all adults when they were asked: to extend novel nouns in labeling phrases (Experiment 1A); to extend novel nouns in labeling phrases with isolated place or object information in the test-trial pictures (Experiment 1B); to find the ones that matched (Experiment 2); or to extend novel nouns in prepositional phrases (Experiment 3). The black dashed line at .50 represents choosing object matches and place matches equally frequently, and the actual proportions of adults’ choices are displayed in white in each bar. Statistical analyses are reported in the text.

All analyses were preregistered prior to data collection. The proportion of object-match responses and place-match responses across all adults in both conditions are shown in Figure 2. The data were analyzed at the individual trial level using three mixed-model binomial logistic regressions, each including participant as a random-effects intercept. To obtain *p*-values, we ran Type 3 Wald tests on the results of each regression, and we also report the models’ estimates as proportions of responses (P) with 95% confidence intervals. The first two intercept-only models tested adults’ choice of object match in each condition against chance. Adults chose significantly more object matches than place matches in the place condition (P = 1.000, 95% CI [.964, 1.000]; *χ2*(1) = 11.57, *p* < .001), but we did not find a difference in their choice of object matches and place matches in the object condition (P = .538, 95% CI [.371, .696]; *χ2*(1) = 0.19, *p* = .663). A third model, which included adults’ responses from both conditions and added a fixed effect for condition, revealed that adults chose more object matches in the place versus object condition (P = .962, 95% CI [.798, .994], *χ2*(1) = 11.60, *p* < .001).

### Discussion

Adults’ responses in the place condition of Experiment 1A suggest that they extend novel nouns to objects more than places. Their performance in the object condition of this experiment shows that this result is specific to a difference in spatial category alone: It cannot be attributed to differences in figure-ground or size relations. These results suggest that adults intuitively think nouns in labeling phrases refer to objects, not places.

One possible alternative explanation for the findings from Experiment 1A, however, is that adults in the place condition were actually extending the novel noun label based on place information but they were doing so by relying on a place’s contents, not its shape. For example, participants might have thought that *blickets* were places that have certain kinds of objects (i.e., of the same shape) in them, as they might think that *kitchen*s are places that have refrigerators in them. Experiment 1B aimed to rule out this possibility by asking adults to extend a novel noun label either to an object-match picture with no place information at all or a place-match picture with no object information at all (Figure 1, Experiment 1B, Place Condition). Regardless of whether places might sometimes be described by an object they contain, object information on its own is unlikely to convey a kind of place (e.g., a refrigerator on its own is unlikely to convey a kitchen). If adults’ responses in the place condition of Experiment 1A were guided by their extending the novel noun label to object information, not to place information via a place’s object contents, then adults in Experiment 1B’s place condition should also extend the novel noun label to object information when no place information is present.

## EXPERIMENT 1B

### Methods

#### Participants.

Thirty-six adults (*M*age: 19 years; range: 18–21 years; 21 women, 15 men) constituted our sample. All were native English speakers, were recruited from our university’s participant pool, and received course credit for their participation. The use of human participants for this experiment was approved by the institutional review board on the use of human subjects at our university. No additional adult participants were excluded.

#### Stimuli and Procedure.

The stimuli and procedure were identical to the place condition of Experiment 1A except that the place-match picture presented just the cartoon person and the same-shaped room as the sample picture and the object-match picture presented just the cartoon person and the same-shaped block as the sample picture (Figure 1, Experiment 1B, Place Condition).

### Results

The data were analyzed as in Experiment 1A. The proportion of object-match responses and place-match responses across all adults are shown in Figure 2. Adults chose significantly more object matches than place matches (P = .998, 95% CI [.749, 1.000]; *χ2*(1) = 5.72, *p* = .017). A second regression including experiment as a fixed effect directly compared the responses from the place condition of Experiment 1A with those of Experiment 1B, and we did not find a significant effect of experiment (P = .402, 95% CI [.037, .922], *χ2*(1) = 0.07, *p* = .787).

### Discussion

Adults in Experiment 1B showed a similar object bias as adults in Experiment 1A, ruling out the possibility that adults in the place condition of Experiment 1A were actually extending the novel noun label based on a place’s object contents. Together, the results in Experiment 1 suggest that adults intuitively think nouns in labeling phrases refer to objects, not places.

Overall, Experiment 1 presented adults with novel noun labels to extend, but this experiment did not examine whether the object bias that adults exhibited was specific to contexts of word learning. As reviewed above, word-learning intuitions and biases about objects, like biases to extend a novel noun to an object of the same shape, are most robustly elicited in young children in contexts in which a noun label is provided. Young children do not show the same shape bias when presented with a sample object and instructed, e.g., “to find one that matches” (Diesendruck & Bloom, 2003; Jones et al., 1991; Landau et al., 1988). Experiment 2 thus explored whether adults might show the same object bias observed in Experiment 1 in the absence of a novel noun label. To address this question, Experiment 2 presented adults with the same stimuli and procedure as Experiment 1, but, rather than asking adults to extend a novel noun, asked adults “to find one that matches.”

## EXPERIMENT 2

### Methods

All of the experiment’s methods were preregistered prior to data collection.

#### Participants.

Seventy-two adults (*M*age: 20 years; range: 18–22 years; 52 women, 19 men, 1 gender nonbinary) constituted our sample, with 36 adults in each of two conditions. All were native English speakers, were recruited from our university’s participant pool, and received course credit for their participation. An additional three adults participated in the experiment but were excluded following preregistered criteria: for taking longer than 15 minutes to complete the experiment (2); or answering at least one catch question incorrectly (1; see below). The use of human participants for this experiment was approved by the institutional review board on the use of human subjects at our university.

#### Stimuli and Procedure.

The stimuli and procedure were identical to Experiment 1A except that participants heard sentences and questions that did not contain novel nouns (Figure 1, Experiment 2). On both trials, the sample picture was accompanied by, “Look! There is a person! And look! Look over here! See that?” When the test pictures appeared, participants were asked, “Where is the one that matches?” For the catch question, participants selected which instructions they had just been given: *find the one that is different*; *find the one with the same name*; *find the one that matches*; *find the one with the same color*; or *something else (Enter what you were asked to do)*.

### Results

All analyses were preregistered prior to data collection. The proportion of object-match responses and place-match responses across all adults in both conditions are shown in Figure 2, and the data were analyzed as in Experiment 1A. Adults chose significantly more object matches than place matches in the place condition (P = 1.000, 95% CI [.992, 1.000]; *χ2*(1) = 16.82, *p* < .001), but we did not find a difference in their choice of object matches and place matches in the object condition (P = .281, 95% CI [.099, .580]; *χ2*(1) = 2.12, *p* = .145). Adults also chose object matches more often in the place versus object condition (P = 1.000, 95% CI [.998, 1.000]; *χ2*(1) = 12.89, *p* < .001).

To examine the effects of the presence or absence of a noun label on adults’ responses, we conducted a fourth regression with condition and experiment (1A versus 2) as fixed effects and found a significant effect of condition (P = .987, 95% CI [.881, .999]; *χ2*(1) = 13.27, *p* < .001), with adults choosing more object matches in the place condition, no effect of experiment (P = .203, 95% CI [.035, .638]; *χ2*(1) = 1.93, *p* = .165), and no condition × experiment interaction (P = .638, 95% CI [.102, .965]; *χ2*(1) = 0.17, *p* = .684). To further evaluate any potential differences across the results of Experiments 1A and 2, we also conducted post hoc paired contrasts between the place condition of both experiments and the object condition of both experiments. We found that neither contrast was significant (place condition: *p* = .414; object condition: *p* = .165).

### Discussion

Adults showed similar responses in Experiments 1A and 2, which differed only by the presence or absence of their being asked to extend a novel noun label. These results suggest that the object bias they demonstrated in Experiment 1A is not specific to word-learning contexts. Rather, their object bias appears to be more general, elicited by language simply asking them to match pictures. Evidently, adults are more likely to both extend the meaning of a novel noun label based on object over place information and match pictures based on object over place information. These results are consistent with prior studies in which adults’ shape bias is present in both word-learning and matching contexts (Landau et al., 1988).

While adults’ object bias may be present beyond word-learning contexts to matching contexts, the results of Experiments 1 and 2 cannot speak to the extent of adults’ object bias across different word-learning contexts with different syntactic framing. In particular, Experiment 1 asked adults to extend novel noun labels, whose syntactic framing may more readily convey object information, compared with, for example, the syntactic framing of novel prepositional phrases, which may more readily convey place information, howsoever it might be defined (Fisher et al., 2006; Landau & Jackendoff, 1993; Landau & Stecker, 1990). In Experiment 3, we thus tested if adults might extend novel nouns to object over place information when those nouns were part of prepositional phrases instead of labeling phrases.

## EXPERIMENT 3

### Methods

All of the experiment’s methods were preregistered prior to data collection.

#### Participants.

Seventy-two adults (*M*age: 20 years; range: 18–23 years; 53 women, 18 men, 1 gender nonbinary) constituted our sample, with 36 adults in each of two conditions. All were native English speakers, were recruited from our university’s participant pool, and received course credit for their participation. An additional 26 adults participated in the experiment but were excluded following preregistered criteria: for taking longer than 15 minutes to complete the experiment (9); or answering at least one catch question incorrectly (17). The greater exclusion rate for this experiment stemmed from adults’ indicating that they heard something else than the novel noun provided, often including some mashup of *acorp* and *blicket*/*wug*. The use of human participants for this experiment was approved by the institutional review board on the use of human subjects at our university.

#### Stimuli and Procedure.

The stimuli and procedure were identical to Experiment 1A except participants heard sentences that contained a novel preposition in addition to a novel noun. After Landau and Stecker (1990), the sample picture was accompanied by, “Look! There is a person! And look! The person is *acorp* a *blicket*! See? She’s *acorp* a *blicket*!” (Figure 1, Experiment 3). The test pictures were accompanied by, “Where is another *blicket*?” All participants heard the first sample picture with the novel noun *blicket* and the second sample picture with the novel noun *wug*.

### Results

All analyses were preregistered prior to data collection. The proportion of object-match responses and place-match responses across all adults in both conditions are shown in Figure 2, and the data were analyzed as in Experiment 1A. Adults chose significantly more object matches than place matches in both the place (P = .728, 95% CI [.574, .842]; *χ2*(1) = 7.92, *p* = .005) and object (P = .705, 95% CI [.530, .834]; *χ2*(1) = 5.17, *p* = .023) conditions. We did not find that adults chose object matches more often in the place versus object condition (P = .556, 95% CI [.345, .748]; *χ2*(1) = 0.26, *p* = .613).

To examine the effects of labeling versus prepositional phrases on adults’ responses, we conducted a fourth regression with condition and experiment (1A versus 3) as fixed effects and found a significant effect of condition (P = .941, 95% CI [.813, .983]; *χ2*(1) = 17.40, *p* < .001), with adults choosing more object matches in the place condition. We did not find a significant effect of experiment (P = .680, 95% CI [.450, .847]; *χ2*(1) = 2.39, *p* = .122). Critically, we also found a significant condition × experiment interaction (P = .074, 95% CI [.016, .284]; *χ2*(1) = 9.57, *p* = .002). Post hoc paired contrasts revealed that adults chose more object matches in the place condition of Experiment 1A (labeling phrases) than in the place condition of Experiment 3 (prepositional phrases; *p* = .005); we did not find any difference in responses in the object condition across experiments (*p* = .122).

### Discussion

Adults showed a different pattern of results in response to novel nouns in prepositional phrases (Experiment 3) compared with novel nouns in labeling phrases (Experiment 1A). Together, the results of Experiments 1A and 3 suggest that a bias to extend novel nouns to objects over places is present in labeling but not prepositional phrases. In particular, and consistent with prior work (Landau & Stecker, 1990), prepositional phrases encouraged more noun extensions to place information, including place information as we define it in the current experiment, as large, extended, and navigable.

Nevertheless, and potentially inconsistent with prior work, our findings in Experiment 3 also suggest that prepositional phrases may encourage a more general figure-over-ground bias since adults in both the place and object conditions extended the novel noun to the block in the foreground of the scene. This result is curious since such a noun in a prepositional phrase is often thought to be the larger and more stable element in the scene (see Landau & Jackendoff, 1993, for a review), and so this result raises several questions. First, prior work on word extension in the context of prepositional phrases has looked at the extension of the novel *prepositions* to place information, not the extension of the novel *nouns* to place information. It is possible that the novel noun following a preposition may most intuitively be what is in the foreground of the scene. Second, in our phrases, the person in the scene was fixed as the subject: “The person is *acorp* a *blicket*.” It is possible that adults found the most intuitive description of the person’s location as relative to something the person might act on, i.e., the block. Third, while the ambiguity of the novel preposition may have made it a better pointer to either object or place information than a novel noun label, it is possible that adults were more likely to interpret the novel preposition as a *particular* preposition that made more sense in the context of the person’s relation to the block (e.g., *near*) than their location relative to the room or container (e.g., *in* or *behind*). Future studies exploring language corpora or examining further the extension of novel nouns following novel prepositions might shed light on these possibilities.

Overall, the results from the adults in Experiments 1–3 nevertheless suggest an object-over-place bias both in the presence and absence of a word-learning context. Within the context of word learning, however, syntactic framing matters, with labeling phrases, not prepositional phrases, privileging object information. In Experiments 4–6, we ask whether 3- and 4-year-old children show similar biases as adults, exploring whether an object bias guides early word learning.

## EXPERIMENT 4A

### Methods

All of the experiment’s methods were preregistered prior to data collection.

#### Participants.

Seventy-two 3- to 4-year-old children (*M*age: 4 years, 0 months; range: 3 years, 0 months–4 years, 11 months; 37 girls, 35 boys) constituted our sample, with 36 children in each of two conditions. All children were typically developing, learning English as a native language, hearing at least 50% English, and were recruited from our lab’s database of families interested in participating in research studies or from a local museum. An additional 10 children participated in the experiment but were excluded following preregistered criteria: for not making a valid response after a maximum of three prompts on either trial (3); not following the instructions (3); experimenter error (3); or interference from caregivers (1). The use of human participants for this experiment was approved by the institutional review board on the use of human subjects at our university.

#### Stimuli and Procedure.

The stimuli were identical to Experiment 1A. The procedure was also identical to Experiment 1A except for the following differences described below. The experiment was presented live and in person with an experimenter in a quiet room at the lab or at the museum instead of remotely on Qualtrics, and the session was video recorded. Prior to starting the experiment, an experimenter instructed caregivers to not interfere with children’s pointing behavior. The experimenter sat next to children and used a mouse to control the stimulus presentation and record children’s responses through PsychoPy, an open-source software for creating and running psychology experiments. The experimenter also produced all the verbal instructions and questions live following a script.

Children first practiced pointing to one of two choices on the screen in four practice trials. For each practice trial, children were shown two black squares, one with a still abstract shape and one with an animated abstract shape. They were asked to point to “where something was happening.” They received positive feedback after each correct response and corrective feedback after each incorrect response. Across the four practice trials, the locations of the correct responses were randomly assigned across children: left, right, right, left; or right, left, left, right.

Children then saw two test trials, as in Experiment 1A. Instead of making a mouse-click response themselves, children were asked to point to the picture on the screen to indicate their response. If children did not point to a picture on the screen after three prompts, their data were excluded (see above) and the experimenter moved on. Children received no feedback on the test trials.

### Results

All analyses were preregistered prior to data collection. The test-trial responses of 9 randomly selected children from each condition (25%) were re-coded by a second coder, masked to children’s condition, using the video recordings. Agreement between the experimenter and the second coder was 100%.

**Figure 3.**
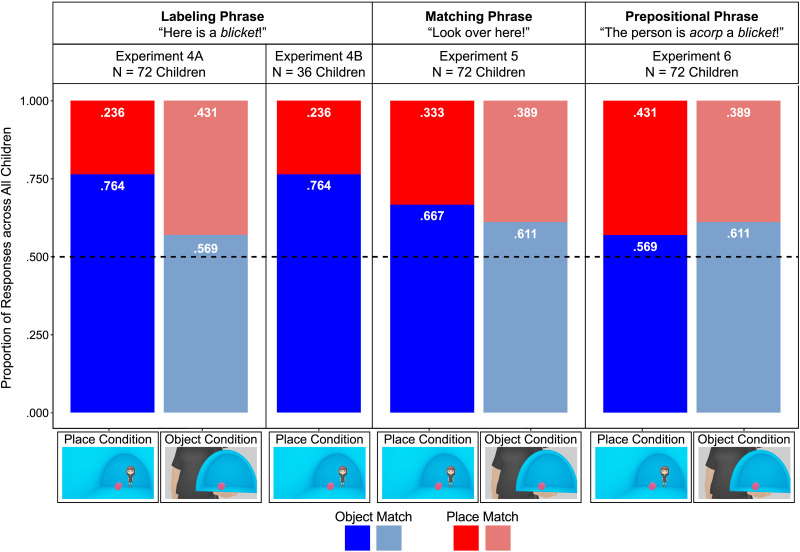
The proportion of object-match responses (in blue) and place-match responses (in red), across all children when they were asked to: extend novel nouns in labeling phrases (Experiment 4A); to extend novel nouns in labeling phrases with isolated place or object information in the test-trial pictures (Experiment 4B); to find the ones that matched (Experiment 5); or to extend novel nouns in prepositional phrases (Experiment 6). The black dashed line at .50 represents choosing object matches and place matches equally frequently, and the actual proportions of children’s choices are displayed in white in each bar. Statistical analyses are reported in the text.

The proportion of object-match responses and place-match responses across all children in both conditions are shown in Figure 3, and the data were analyzed as in Experiment 1A. Children chose significantly more object matches than place matches in the place condition (P = .764, 95% CI [.653, .848]; *χ2*(1) = 17.90, *p* < .001), but we did not find a difference in their choice of object matches and place matches in the object condition (P = .601, 95% CI [.414, .762]; *χ2*(1) = 1.13, *p* = .289). Children also chose object matches more often in the place versus object condition (P = .729, 95% CI [.533, .864]; *χ2*(1) = 5.14, *p* = .023).

To examine any effects of age within the child sample, we conducted a fourth regression including condition and age (treated as a continuous variable and mean-centered) as fixed effects and found a significant effect of condition (P = .727, 95% CI [.533, .861]; *χ2*(1) = 5.11, *p* = .024), with children choosing more object matches in the place condition. We did not find an effect of age (P = .634, 95% CI [.490, .757]; *χ2*(1) = 3.35, *p* = .067) or a condition × age interaction (P = .402, 95% CI [.226, .608]; *χ2*(1) = 0.86, *p* = .352).

Finally, to examine any differences between the adult and child samples tested across Experiments 1A and 4A, we conducted a fifth regression including condition and age group (treated as a categorical variable: adults versus children) as fixed effects. Here, there was a significant effect of condition (P = .937, 95% CI [.809, .981]; *χ2*(1) = 17.79, *p* < .001), with participants choosing more object matches in the place condition, but no effect of age group (P = .553, 95% CI [.338, .751]; *χ2*(1) = 0.22, *p* = .638). In addition, there was a significant condition × age group interaction (P = .167, 95% CI [.043, .473]; *χ2*(1) = 4.43, *p* = .035). While both children and adults chose the object matches more in the place condition, this difference was larger for adults.

### Discussion

Children’s responses in the place condition of Experiment 4A suggest that they, like adults, extend novel nouns to objects more than places and that this result is specific to a difference in spatial category alone. Moreover, while the strength of this effect did not change from 3 to 4 years of age, adults did show this object bias more strongly than did young children. These results are consistent with the developmental strengthening of other word-learning biases about objects, like the shape bias (Landau et al., 1988).

To address the possibility that children in the place condition of Experiment 4A were extending the novel noun label based on place contents, we conducted Experiment 4B, which was modeled after Experiment 1B with adults. Experiment 4B followed the same procedure as Experiment 4A except it included object-match pictures with no place information and place-match pictures with no object information at all (Figure 1, Experiment 4B).

## EXPERIMENT 4B

### Methods

#### Participants.

Thirty-six 3- to 4-year-old children (*M*age: 3 years, 11 months; range: 3 years, 0 months–4 years, 11 months; 20 girls, 16 boys) constituted our sample. All were typically developing, learning English as a native language, hearing at least 50% English, and were recruited from our lab’s database of families interested in participating in research studies or from a local museum. An additional eight children participated in the experiment but were excluded following criteria: for not completing the experiment (1); not following the instructions (1); not making a valid response after a maximum of three prompts on either trial (3); or experimenter error (3). The use of human participants for this experiment was approved by the institutional review board on the use of human subjects at our university.

#### Stimuli and Procedure.

The stimuli and procedure were identical to the place condition of Experiment 4A except the object-match and place-match pictures were those used in Experiment 1B.

### Results

The data were analyzed as in Experiment 4A. The test-trial responses of 9 randomly selected children (25%) were re-coded by a second coder, masked to children’s condition, using the video recordings. Agreement between the experimenter and the second coder was 100%.

The proportion of object-match responses and place-match responses across all children are shown in Figure 3, and the data were analyzed as in the place condition of Experiment 4A. Children chose significantly more object matches than place matches (P = 1.000, 95% CI [.984, 1.000]; *χ2*(1) = 16.69, *p* < .001). A second model including age (treated as a continuous variable and mean-centered) found no effect of age on children’s responses (P = .735, 95% CI [.153, .977]; *χ2*(1) = 0.54, *p* = .464). A third regression including experiment as a fixed effect directly compared the responses from the place condition of Experiment 4A with those of Experiment 4B, and we did not find a significant effect of experiment (P = .510, 95% CI [.270, .746]; *χ2*(1) = 0.01, *p* = .938). Finally, to examine any differences between the adult and child samples tested across Experiments 1B and 4B, we conducted a fourth regression including age group (treated as a categorical variable: adults versus children) as a fixed effect; we did not find a significant effect of age group (P = .268, 95% CI [.018, .877]; *χ2*(1) = 0.44, *p* = .506).

### Discussion

Children in Experiment 4B showed a similar object bias as children in Experiment 4A and ruled out the possibility that children in the place condition of Experiment 4A were actually extending the novel noun label based on a place’s object contents. Like adults, children intuitively think nouns in labeling phrases refer to objects, not places.

In the prior work described above, children, unlike adults, however, did not show word-learning biases, like the shape bias, outside of word-learning contexts, e.g., when presented with a sample object and instructed “to find one that matches” (Diesendruck & Bloom, 2003; Jones et al., 1991; Landau et al., 1988). Modeled after Experiment 2 with adults, Experiment 5 thus examined if children show the same object bias as in Experiment 4A when they were asked to match pictures.

## EXPERIMENT 5

### Methods

All of the experiment’s methods were preregistered prior to data collection.

#### Participants.

Seventy-two 3- to 4-year-old children (*M*age: 4 years, 0 months; range: 3 years, 1 month–4 years, 11 months; 36 girls, 36 boys) constituted our sample, with 36 children in each of two conditions. All children were typically developing, learning English as a native language, hearing at least 50% English, and were recruited from our lab’s database of families interested in participating in research studies or from a local museum. An additional 5 children participated in the experiment but were excluded following preregistered criteria: for not making a valid response after a maximum of three prompts on either trial (4); or interference from caregivers (1). The use of human participants for this experiment was approved by the institutional review board on the use of human subjects at our university.

#### Stimuli and Procedure.

The stimuli and procedure were identical to Experiment 4A except that, as in Experiment 2 with adults, children heard sentences and questions that did not contain novel nouns. They were simply asked “to find the one that matches” (Figure 1, Experiment 5).

### Results

All analyses were preregistered prior to data collection. The test-trial choices of 9 randomly selected children from each condition (25%) were re-coded by a second coder, masked to children’s condition, using the video recordings. Agreement between the experimenter and the second coder was 100%.

The proportion of object-match responses and place-match responses across all children in both conditions are shown in Figure 3, and the data were analyzed as in Experiment 4A. Children chose significantly more object matches than place matches in both the place (P = .999, 95% CI [.952, 1.000]; *χ2*(1) = 11.71, *p* < .001) and object condition (P = 1.000, 95% CI [.988, 1.000]; *χ2*(1) = 18.19, *p* < .001). We did not find a difference in children’s choice of the object versus place matches across conditions (P = .581, 95% CI [.060, .968]; *χ2*(1) = 0.04, *p* = .836).

The model including condition and age (treated as a continuous variable and mean-centered) within the child sample revealed no significant effect of condition (P = .934, 95% CI [.140, .999]; *χ2*(1) = 1.36, p = .244), but a significant effect of age (P = .001, 95% CI [.000, .167]; χ2(1) = 6.59, *p* = .010), with younger children choosing more object matches, and a significant condition × age interaction (P = .999, 95% CI [.857, 1.000]; *χ2*(1) = 6.69, *p* = .010). Using a planned simple slopes analysis, we found no difference in older versus younger children’s choice of object matches in the place condition (*p* = .638), but older versus younger children chose more object matches in the object condition (*p* = .010). The model including condition and age group (now treated as a three-level categorical variable, which was specified in the preregistration given the age effects in the previous model) across the adult and child samples tested in Experiments 2 and 5 revealed significant effects of condition (P = 1.000, 95% CI [.999, 1.000]; *χ2*(1) = 16.02, *p* < .001), age group (*χ2*(2) = 12.39, *p* = .002), and a significant condition × age group interaction (*χ2*(2) = 16.09, *p* < .001). Planned Holm-corrected pairwise contrasts compared the performance of each group directly. In the place condition, we found no differences between any of the age groups (3-year-olds versus adults: *p* = .850; 4-year-olds versus adults: *p* = 1.000; 3-year-olds versus 4-year-olds: *p* = 1.000). In the object condition, adults chose more place matches than 3-year-olds (*p* = .001), but we did not find a difference between the responses of adults and 4-year-olds (*p* = .629) or 3-year-olds and 4-year-olds (*p* = .629).

Finally, to examine the effects of the presence or absence of a noun label on children’s responses, we evaluated the effects of condition and experiment (4A versus 5) as fixed effects and found a significant effect of condition (P = .802, 95% CI [.516, .939]; *χ2*(1) = 4.21, *p* = .040), with children choosing more object matches in the place condition, no effect of experiment (P = .581, 95% CI [.278, .833]; *χ2*(1) = 0.25, *p* = .618), and no condition × experiment interaction (P = .270, 95% CI [.055, .701]; *χ2*(1) = 1.12, *p* = .291). To further evaluate any potential differences across the results of Experiments 4A and 5, we also conducted post hoc paired contrasts between the place condition of both experiments and the object condition of both experiments. We did not find that either contrast was significant (place condition: *p* = .320; object condition: *p* = .618).

### Discussion

Experiments 4A and 5 differed only by children being asked to either extend a novel noun label or match pictures. There were two important findings in children’s responses in Experiment 5 that were different from those of Experiment 4A. In Experiment 5, children chose significantly more object matches in the object condition compared with chance; and we did not find a difference in children’s object-match responses across the place and object conditions. These results may suggest, albeit weakly given the lack of a significant interaction between the experiments, that the word-learning context in Experiment 4A bolstered children’s object bias. Children’s tendency in Experiment 5 to choose the object match in both conditions in the matching context may suggest that this matching context favors a kind of extension that focuses more on figure information in general rather than on object information in particular.

In addition to these findings, the significant interaction between adults’ performance in the matching context of Experiment 2 compared with children’s performance in the matching context of Experiment 5 suggests that there is developmental change in how word-learning versus matching contexts focuses participants on object information, with adults adopting an object bias even outside of word-learning contexts more readily than young children. These results are consistent with the strengthening of other word-learning biases, like the shape bias, with age (Landau et al., 1988).

Children’s object bias is present in word-learning contexts presenting novel noun labels, whose syntactic framing may readily convey object information. In Experiment 6, we ask if children’s object bias is present when they are given novel prepositional phrases, whose syntactic framing may more readily convey place information, howsoever it might be defined (Fisher et al., 2006; Landau & Jackendoff, 1993; Landau & Stecker, 1990). Experiment 6 is modeled after Experiment 3 with adults, who chose more place matches when extending novel nouns in prepositional versus labeling phrases.

## EXPERIMENT 6

### Methods

All of the experiment’s methods were preregistered prior to data collection.

#### Participants.

Seventy-two 3- to 4-year-old children (*M*age: 4 years, 0 months; range: 3 years, 0 month–4 years, 11 months; 39 girls, 33 boys) constituted our sample, with 36 children in each of two conditions. All children were typically developing, learning English as a native language, hearing at least 50% English, and were recruited from our lab’s database of families interested in participating in research studies or from a local museum. An additional eight children participated in the experiment but were excluded following preregistered criteria: for not completing the study (2); not following the instructions (1); not making a valid response after a maximum of three prompts on either trial (2); or experimenter error (3). The use of human participants for this experiment was approved by the institutional review board on the use of human subjects at our university.

#### Stimuli and Procedure.

The stimuli and procedure were identical to Experiment 4A with children except that, as in Experiment 3 with adults, children heard sentences that contained a novel preposition in addition to a novel noun.

### Results

All analyses were preregistered prior to data collection. The test-trial choices of 9 randomly selected children from each condition (25%) were re-coded by a second coder, masked to children’s condition, using the video recordings. Agreement between the experimenter and the second coder was 100%.

The proportion of object-match responses and place-match responses across all children in both conditions are shown in Figure 3, and the data were analyzed as in Experiment 4A. We did not find that children chose either kind of match more often in either the place (P = .601, 95% CI [.414, .762]; *χ2*(1) = 1.13, *p* = .289) or object (P = .626, 95% CI [.481, .752]; *χ2*(1) = 2.92, *p* = .087) condition. We also did not find that children’s responses differed by condition (P = .446, 95% CI [.247, .665]; *χ2*(1) = 0.22, *p* = .639).

The model including condition and age (treated as a continuous variable and mean-centered) within the child sample revealed no significant effects of condition (P = .438, 95% CI [.239, .660]; *χ2*(1) = 0.28, *p* = .594), age (P = .506, 95% CI [.347, .664]; *χ2*(1) = 0.01, *p* = .943), or a condition × age interaction (P = .540, 95% CI [.319, .746]; *χ2*(1) = 0.12, *p* = .731). In the model including condition and age group (treated as a categorical variable: adults versus children) as fixed effects, we did not find significant effects of condition (P = .557, 95% CI [.338, .756]; *χ2*(1) = 0.25, *p* = .616), age group (P = .427, 95% CI [.236, .643]; *χ2*(1) = 0.43, *p* = .513), or a condition × age group interaction (P = .392, 95% CI [.156, .693]; *χ2*(1) = 0.47, *p* = .491).

Finally, to examine the effects of hearing labeling phrases versus prepositional phrases on children’s responses, we evaluated the effects of condition and experiment (4A versus 6) as fixed effects and found a significant effect of condition (P = .739, 95% CI [.535, .875]; *χ2*(1) = 5.13, *p* = .024), with children choosing more object matches in the place condition, but no effect of experiment (P = .551, 95% CI [.346, .740]; *χ2*(1) = 0.23, *p* = .634). Critically, we also found a significant condition × experiment interaction (P = .223, 95% CI [.077, .498]; *χ2*(1) = 3.89, *p* = .049). To further evaluate the differences across experiments, we conducted post hoc paired contrasts between the place condition of both experiments and the object condition of both experiments. Children chose more object matches in the place condition of Experiment 4A versus Experiment 6 (*p* = .024), but we did not find any difference in children’s responses in the object condition across experiments (*p* = .634).

### Discussion

Like adults, children showed a different pattern of results in response to novel nouns in prepositional phrases (Experiment 6) than they did in response to novel nouns in labeling phrases (Experiment 3): Children’s bias to extend novel nouns to objects over places was present in their responses to labeling phrases but not to prepositional phrases. These results are consistent with prior work, in which young children show sensitivity to syntactic framing and prepositional phrases in word-learning contexts (Fisher et al., 2006; Landau et al., 1992; Landau & Stecker, 1990; White & Lidz, 2022).

While children in Experiment 6, like adults in Experiment 3, appeared to show an overall preference across conditions to extend the novel noun to the figure information (i.e., the block) over the ground information (i.e., the room or container), it is nevertheless hard to make strong claims about this effect. Unlike adults’ responses in Experiment 3, children’s responses in neither the place nor object condition of Experiment 6 alone showed a significant effect of figure information; indeed, we did not find in either condition that children’s responses were different from chance. And, there was no option of a third picture, matching the sample in neither figure nor ground information that could have indicated whether children might have been choosing randomly in this experiment (Kenderla et al., 2023). While prior work has found that children as young as 19 months of age can understand phrases with two novel words and can even assign different meanings to a novel noun when it appears either as the direct object of a novel verb (e.g., “She’s *meeking* the *tiv.*”) or as the instrument of a novel verb (e.g., “She’s *meeking* with the *tiv*.”; White & Lidz, 2022), young children in the present study may have either found our novel prepositional phrases too difficult to understand or they might not yet have strong biases about such a noun’s extension to object, place, figure, or ground information. Relatedly, children may have found the choice between the two possible matches lacking in the information they were looking for. In particular, if children were looking to extend the novel noun based on the person’s *location* relative to anything in the scene, as might be suggested by prior work on the language of spatial relations (e.g., Landau & Jackendoff, 1993), they would have found no discriminating location information to choose from: Both the place- and object-match pictures presented the same relative location information between the person and the other elements in the scene. The place- and object-match pictures varied only by their shape information, which, as Landau and Jackendoff (1993) outline, might be disregarded when specifying roles in a spatial relation lexicalized by a preposition. While adults may have been able to understand our novel prepositional phrases in the context of the varying shape information, future work with adults and children might explore how participants would respond when the position of the person is also varied relative to the room, the container, or the block, examining interactions among objects, places, and locations.

## GENERAL DISCUSSION

In Experiments 1 and 4, we found that adults and young children show an object-over-place bias in word learning. They preferentially extend novel noun labels to object information over place information, and this happens when the object and place information includes diagnostic, core geometric features (e.g., bounded shape versus navigable layout) and is dissociated from more general figure-ground and size relations. We also tested the specificity of this bias to a word-learning context by evaluating in Experiments 2 and 5 whether the same bias was present in a simple matching context. We found that adults demonstrated the same object bias in both word-learning and matching contexts. While young children demonstrated the object bias in the word-learning context, their responses showed a less clear pattern for the matching context. Finally, in Experiments 3 and 6, we tested whether adults and children also showed an object bias in a different word-learning context—when extending novel nouns in novel prepositional phrases—since extending the meaning of a novel noun in a prepositional phrase may more readily point to place information, howsoever it is defined. We found that adults’ and children’s object bias was not present when extending novel nouns following prepositions.

In extending novel noun labels, spatial category information from at least young childhood is thus used as a foundational source of information with inherent biases, apart from any biases rooted in more general, nondomain-specific spatial information like figure-ground or size relations. This use of spatial category information may apply to symbolic contexts more generally, including to both linguistic and pictorial contexts, as suggested by the object-over-place bias in young children’s drawings (Dillon, 2021).

Nevertheless, the object bias in language observed in the present study has particular scope and limits, which may change through development in a way that is similar to that of other word-learning biases about objects, like the shape bias. In particular, adults show a stronger object bias than children do and a generalization of the bias outside of word-learning contexts to matching contexts, a generalization children do not yet have. Both adults and children, moreover, demonstrate limits to this bias within a word-learning context since the bias is not present when they extend the meaning of a novel noun in a novel prepositional phrase.

Adults and children’s object bias in extending the meaning of novel noun labels may have a variety of nonmutually exclusive sources such as linguistic experience, nonlinguistic experience, or pre-existing, early emerging knowledge about spatial domains like objects and places shared by humans and nonhuman animals alike.

In terms of linguistic experience, for example, Sandhofer et al. (2000) found that of the 43 most common nouns in the CHILDES corpora (MacWhinney, 2000), 74% of them referred to objects, and Frank et al. (2021) found in the Wordbank project’s database of MacArthur Bates Communicative Development Inventories norming data that place nouns are underrepresented cross-linguistically relative to nouns that refer to other semantic categories, like body parts, vehicles, animals, and toys. Moreover, Lin and Dillon (2023) found that 12-month-old infants understand more object nouns than place nouns, as reported in the MacArthur Bates Communicative Development Inventories norming data (Frank et al., 2017), and, among the utterances produced by caregivers in the CHILDES corpora, object nouns occurred more often than place nouns, and phrases with object versus place nouns were qualitatively different: Caregivers were more likely to label objects (e.g., “It’s a *ball*.”) than places, but were more likely to talk about places than objects in the context of navigation and with prepositional phrases (e.g., “Go to the *kitchen*.”; “They’re at the *beach*.”). The present findings partially mirror these patterns. In particular, differences in object nouns’ and place nouns’ frequency and context of occurrence in early linguistic experience may encourage adults and children to expect novel noun labels to refer to objects over places. Nevertheless, the greater use of place nouns in prepositional phrases in linguistic experience does not fully reflect adults’ and children’s performance in the present experiments since we did not find that they preferentially extended novel nouns in prepositional phrases to places.

Another possible source of the object bias in the present experiments is early nonlinguistic experience. While infants manipulate objects in early infancy (Rochat, 1989), they do not move through places on their own until much later (Adolph & Franchak, 2017). In addition, younger infants’ line of sight limits their view of walls and other features of the extended surface layout composing a place and accentuates their view of objects (Kretch et al., 2014; Smith et al., 2018; Soska et al., 2010). Infants’ visual experience based on their postural development, moreover, shapes their language learning (Libertus & Violi, 2016; Walle & Campos, 2014; Yu & Smith, 2012). Although this nonlinguistic experience cannot explain the difference in the object bias across labeling and prepositional phrases, infants’ object-focused visual experience may nevertheless contribute to their object bias in word learning more generally (Smith, 2003; Smith et al., 2002).

Finally, foundational differences in the way not only children, but also adults and nonhuman animals interact with objects and places for everyday recognition and navigation might affect young children’s intuitions about which spatial category language is more likely to pick out (Landau, 2017; Landau & Jackendoff, 1993), leading to an object bias. In particular, while humans and nonhuman animals tend to use geometry, like the allocentric distances and directions of the boundaries of the large-scale environment, automatically and implicitly to determine their position in space (Cheng & Gallistel, 1984; Cheng & Newcombe, 2005; Hermer & Spelke, 1994, 1996; Spelke & Lee, 2012), they tend to learn their position in space deliberately and explicitly relative to the location of landmark objects of specific shapes (e.g., Barry & Muller, 2011; Doeller & Burgess, 2008; Doeller et al., 2008; Shusterman et al., 2011; Twyman et al., 2007). Children and adults may thus expect that labeling language, which is an explicit and intentional communicative tool, is more likely to pick out objects. While there was no actual navigation in the present study’s tasks (participants simply viewed 2D pictures of places and objects), the present study may have nevertheless tapped into these foundational spatial-domain distinctions, connecting the explicitness of object representations to the explicitness of expressed language.

## ACKNOWLEDGMENTS

We thank the participating families at the lab and at the National Museum of Mathematics for volunteering their time.

## FUNDING INFORMATION

This work was supported by a National Science Foundation CAREER Award (DRL-1845924; to M. R. D.).

## AUTHOR CONTRIBUTIONS

Both authors designed the study. Y. L. created the stimuli and collected and analyzed the data. Both authors wrote the manuscript.

## DATA AVAILABILITY STATEMENT

All materials, methods, preregistrations, data, and analyses are available at: https://osf.io/v46bu/.
